# MEG correlates of speech planning in simple vs. interactive picture naming in children and adults

**DOI:** 10.1371/journal.pone.0292316

**Published:** 2023-10-17

**Authors:** Ebony Goldman, Sherine Bou-Dargham, Marco Lai, Anvita Guda, Jacqui Fallon, Miriam Hauptman, Alejandra Reinoso, Sarah Phillips, Ellie Abrams, Alicia Parrish, Liina Pylkkänen

**Affiliations:** 1 Department of Psychology, New York University, New York, NY, United States of America; 2 NYUAD Research Institute, New York University Abu Dhabi, Abu Dhabi, UAE; 3 Department of Linguistics, New York University, New York, NY, United States of America; 4 Department of Psychology, Johns Hopkins University, Baltimore, MD, United States of America; 5 Department of Communication Sciences and Disorders, Northwestern University, Evanston, IL, United States of America; 6 Center for Brain Plasticity and Recovery, Georgetown University, Washington, DC, United States of America; Basque Center on Cognition Brain and Language, SPAIN

## Abstract

The picture naming task is common both as a clinical task and as a method to study the neural bases of speech production in the healthy brain. However, this task is not reflective of most naturally occurring productions, which tend to happen within a context, typically in dialogue in response to someone else’s production. How the brain basis of the classic “confrontation picture naming” task compares to the planning of utterances in dialogue is not known. Here we used magnetoencephalography (MEG) to measure neural activity associated with language production using the classic picture naming task as well as a minimal variant of the task, intended as more interactive or dialogue-like. We assessed how neural activity is affected by the interactive context in children, teenagers, and adults. The general pattern was that in adults, the interactive task elicited a robust sustained increase of activity in frontal and temporal cortices bilaterally, as compared to simple picture naming. This increase was present only in the left hemisphere in teenagers and was absent in children, who, in fact, showed the reverse effect. Thus our findings suggest a robustly bilateral neural basis for the coordination of interaction and a very slow developmental timeline for this network.

## Introduction

Although human language is both an action and a perception system—we produce language that others subsequently comprehend—our understanding of the neural underpinnings of the action part is grossly underdeveloped as compared to the perception part. Why is this? The reasons are mostly methodological. It is easy to conduct experiments that target different stages of comprehension by delivering different types of stimuli to study participants. Even nonwords like *yuuk*, which has phonology but no meaning, can serve as stimuli. Parallel production studies would have participants produce different types of linguistic expressions. All participants would need do this exactly in the same way, in order to mimic comprehension studies in which everyone gets the same stimuli. But the precise way that people talk and describe things is hard to control.

The standard approach to the consistency challenge has been the “simple” picture naming task. In this task, participants are shown carefully chosen pictures that are likely to be named in a highly consistent way. The consistency is usually achieved because the pictures have been pre-tested to elicit consistent naming or because the subjects are explicitly instructed to name the pictures in a certain way. Most commonly, the pictures depict straightforwardly nameable objects, but sometimes they may depict more complex scenes or actions. The more complex the intended linguistic descriptions, the harder it is to achieve naming consistency. Nevertheless, the picture naming task is the gold-standard in production research, including in clinical practice [1]. But it clearly only represents one, somewhat narrow aspect of language production: contextless description of objects and events in our environment. The most natural instance of this may be child-directed speech: “Look, apple!” But at least intuitively, a much larger proportion of naturally occurring productions are responses to another person’s utterance. It is those situations that we aimed to model in the current work, albeit with a task that is still very much a laboratory task.

We created an interactive task by modifying the classic picture naming task only slightly. Instead of one picture, the subjects were shown two pictures, say a cup and a house. The computer named one of the pictures and then the subject’s task was to name the other. While obviously an unnatural laboratory task, this set-up accomplishes a few useful modifications to aid our understanding of naturalistic speech production. First, like natural dialogue, it engages the full comprehension-to-production system: subject hears a description and plans a response based on it. Second, like the classic picture naming task, the utterances are fully controlled. We used a small set of objects and had the subjects practice naming them in the intended way. Third, the mini-dialogues were conducted not just with the object names, but also with color-shape descriptions, as in “green cup–blue house,” engaging both word- and phrase-level representations, both in comprehension and production. Thus, a fairly simple and highly controlled task managed to drive both the retrieval and combinatory aspects of language in an interactive setting. Given its simplicity, the task is highly suited for many different types of populations. Here, we report what neural circuits it engages as compared to simple picture naming in adults, teens and school-age children.

As compared to simple picture naming, our interactive task contained components that could both decrease and increase neural activity. On the one hand, the utterances in the interactive task occurred in a highly facilitatory context: the initial picture of the two objects and the subsequent naming of one of them by the computer allowed participants to begin planning their utterances already during the computer’s speech, similar to natural conversation. This predicts faster response times for the interactive than the simple naming task as a function of contextual support, potentially correlating with reduced neural activity. On the other hand, interaction involves more mental faculties than simple picture naming, including representing the interlocutor’s mental states, turn-taking and interactional synchrony [2]. Prior work has indicated that the right hemisphere (RH) may contribute importantly to aspects of conversation, such as turn-taking [3–6]. Areas of the RH also activate more strongly for the comprehension of dialogue than monologue [7]. Predictive processing, in contrast, is most likely highly bilateral, with somewhat different models of prediction operating in the two hemispheres [8]. Our MEG analyses tested for interaction related activity increases and decreases and characterized how they emerged during development.

### Neural bases of classic picture naming in adults and children

A large prior literature has used functional magnetic resonance imaging (fMRI) and positron emission tomography (PET) to investigate the neural bases of single word production, most commonly with the picture-naming task. The task provides insight into multiple components of normal language production, namely semantic access, lexical access, motor planning, and articulation [9, 10]. The hemodynamic literature has implicated a wide network of areas for the production of single words: left superior temporal gyrus (STG), left primary motor cortex, left premotor cortex [11, 12], supplementary motor area (SMA), insula, thalamus, basal ganglia and posterior cerebellum [13], and inferior frontal gyri (IFG) [12, 14]. The spatiotemporal dynamics of word production have also been characterized with magnetoencephalography (MEG), which has revealed involvement of the left posterior temporal lobe/Wernicke’s area, angular gyrus and Broca’s area [15], as well as the right parietal cortex [16], with activation starting from the occipital cortex at around 200 ms and then proceeding to temporal and parietal regions [15–19]. More recently though, a much more parallel organization has emerged from electroencephalography (EEG) using the same task [20]. Beyond the single word level, the planning of combinatory phrases in language production has elicited increased activity in left anterior temporal and ventromedial prefrontal cortices as compared to non-combinatory controls [21, 22], paralleling findings from comprehension [23]. Here we also used MEG, aiming to utilize its spatial and temporal resolution to assess cortical differences between confrontation picture naming and our novel interactive version of the task.

While there exists a coherent body of work on the neural correlates of picture naming in adults, the findings for children are more disparate, and mostly come from studies on clinical populations, such as children who stutter [24, 25] or children with specific language impairments [26, 27], brain lesions [28] or epilepsy [29]. While these studies commonly use typically developing children as controls, their primary focus is not healthy children, given that the main question lies elsewhere. Some commonly implicated cortical areas of activity associated with picture naming task in healthy children include the left IFG [26] and Broca’s area [29]. Still, the functional network involved in picture naming has not yet been characterized as clearly in children as it has been in adults. In electrophysiology, Atanasova et al. [30] used event-related potentials (ERPs) to investigate developmental changes in picture naming, finding that by 17 years of age, neural responses were almost completely adult-like, with 13–16-year-olds showing an intermediate, somewhat adult-like pattern. According to these results, the shift into adult-like activation begins around 13 years of age.

### Prior work addressing the neural basis of interaction/dialogue

The brain basis of interactive dialogue or conversation has been investigated much less than that of simple picture naming. Natural dialogue crucially involves joint attention and social cooperation between conversation partners. The prefrontal cortex (PFC) and specifically the medial prefrontal cortex (mPFC), and ventromedial prefrontal cortex (vmPFC) are consistently implicated in studies where participants are cooperating in a social context with others, a necessary aspect of dialogue [31, 32]. Similarly, Gordon et al. [33] suggest that the vmPFC is a critical part in synchronizing social behavior in a conversational context.

Neuroscience research on dialogue and language production in conversation has also commonly assessed phenomena such as neural synchronization and coupling between interlocutors. We can ask how brain activity is modulated by the knowledge that one is conversing with a partner. Prior work investigating synchrony during conversation implicates the left posterior STG [34], the posterior superior temporal sulcus (pSTS), and temporo-parietal junction (TPJ) [35]. Investigations into dialogue and conversation have also addressed how speech planning and production in dialogue are represented in motor cortex, proposing that although motor cortex is not essential for the perception of speech, it is essential for conversation, governing turn-taking and interactional synchrony [2].

The child literature on dialogue has also commonly focused on the phenomenon of neural synchrony in conversation, specifically with adult interlocutors, such as parents, educators, and caretakers [36–39]. While these studies tell us a lot about how social interaction affects the neurocognitive mechanisms of language production, they do not identify specific brain regions responsible for these phenomena.

In summary, there is little research on the specific mechanisms of cortical activity in a dialogue context in children. Additionally, no studies as of yet have investigated how cortical activity differs between confrontation naming and dialogue in adults or in children. The current work takes the first step towards filling this gap by investigating an interactive version of the classic picture naming task across three age groups, children, teenagers, and adults.

## Methods

### Participants

Fifty English speakers took part in this study. Recruitment was severely challenged by the Covid-19 pandemic. Participants were separated into three age categories: (1) Adults (n = 17, 10 females, 7 males, *M*_*age*_ = 35, *SD* = 13.9, 17 right-handed, 0 left-handed), (2) Teenagers (n = 15, 12 females, 3 males, *M*_*age*_ = 18, *SD* = 1.3, 14 right-handed, 1 left-handed) and (3) Children (n = 18, 8 females, 10 males, *M*_*ag*e_ = 9, *SD* = 1.6, 17 right-handed, 1 left-handed). All participants were proficient English speakers with no history of neurological disorders. All participants had healthy or corrected-to-healthy vision and healthy hearing. All adult participants and the parents of all child and teenage participants provided informed written consent following NYU International Review Board specifications. Participants received either monetary compensation or course credit for completing the study.

### Design

**Fig 1:**
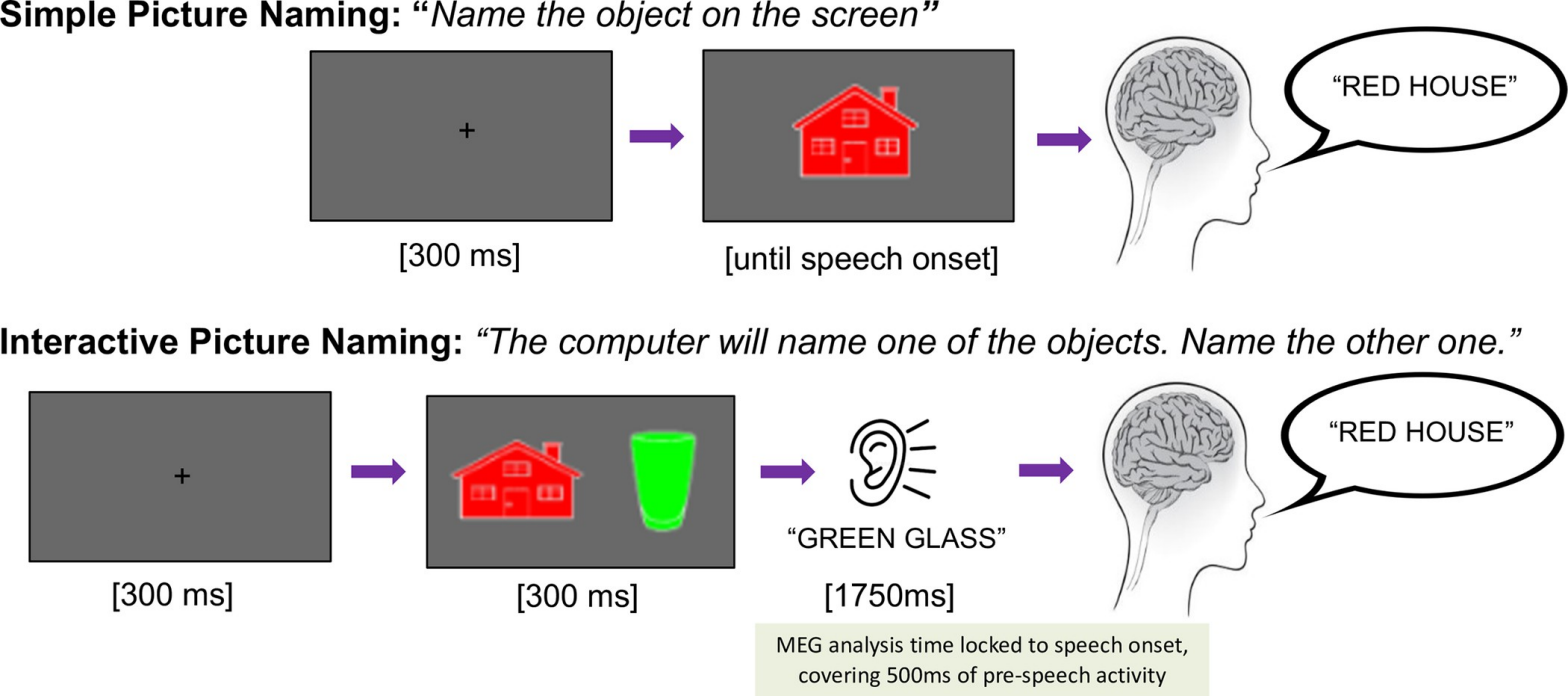
Task design and trial structure. In all trials in both tasks, participants were asked to name an object on the screen. One task was the simple picture naming task, where the participants were asked to say the name of the object that was shown on the screen. The picture stayed on the screen until speech onset, to avoid any visual offset response within the pre-speech analysis window (as in Pylkkänen et al. [22]). In the other task, the interactive picture naming task, participants first saw two objects on the screen for 300ms as the interactive context. The pictorial context then disappeared to eliminate eye movements around the more complex image, and immediately at the offset of the image, participants heard the computer speak the name of one of depicted objects. The participants’ task was to name the other object, the one the computer did not name.

Our main aim was to compare simple picture-naming with our novel interactive picture-naming task (Fig 1). In simple picture naming, participants saw a colored image on the screen and were asked to name the object that they saw. In interactive picture naming, two images were presented, the computer named one of them, and then the participant’s task was to name the other image. This captured the core stages of interaction: comprehending an utterance and then then planning a response accordingly. In the interactive task, participants were instructed to not interrupt or talk over their interlocutor, that is, the computer.

These two tasks were presented in the context of a larger multi-task protocol designed to measure neural correlates associated with language comprehension and production at the lexical and phrasal levels in adults and children. The simple and interactive picture naming tasks also involved single word and phrase blocks, but this manipulation did not affect activity time-locked to production onset, which is our focus here, and thus we collapse across the two stimulus types. Both tasks employed a blocked design. For each task, there were 6 blocks in the experiment, 3 of which were the phrase condition and 3 of which were the single-word conditions. Participants also took part in a baseline task involving no productions, but this task could not be included in a production locked analysis, and thus is excluded from the current report. The order of the blocks was randomized across participants.

The stimuli used across these tasks were 6 objects (comb, door, glass, heart, house, sword) displayed in 6 colors (black, blue, green, pink, red, white), both matched for phoneme number (colors: *M* = 4.33, *SD* = 0.75; nouns: *M* = 4.67, *SD* = 0.47; *p* = 0.47, paired t-test), log HAL frequency (colors: *M* = 11.3, *SD* = 0.84; nouns: *M* = 10.0, *SD* = 1.33; *p* = 0.19, paired t-test) [40], and age of acquisition scores (colors: mean = 3.74 years, sd = 0.18; nouns: *M* = 4.47, *SD* = 1.02; *p* = 0.14, paired t-test) [41]. We selected objects that children likely encounter in their daily lives to ensure high production accuracy. An additional constraint was that the same study was being planned in Arabic, and thus we chose items that also worked in Arabic. Instructions at the beginning of the block differentiated the type of utterance that the participant was expected to produce. Since the phrasal vs. single-word production tasks used the same stimuli but different instructions, each task was presented in separate blocks. Each block contained a randomized subset (N = 25) of all the possible object and color combinations. All images were presented foveally using Presentation software (Neurobehavioral Systems) and subtended in a range from 1.65° height and 2.55° width on a screen ≈ 85 cm from the subject. The size of the picture ensured that only one fixation was required to perceive all of the elements of the stimuli, allowing us to avoid the emergence of saccade-related artifacts.

The auditory stimuli for the dialogue task were recorded by a female native English speaker. Audio files were not stretched to create a maximally naturalistic auditory experience (so they were not all the same length). To ensure that all epochs were the same length and the onset of each adjective and noun was the same across trials, we added silence to the end of each individual word (length of silence was dependent on how long the speech lasted) and all audio files were scaled to 70 dB.

### Procedure

Before the MEG recordings, each participant’s head shape was digitized using a Polhemus dual source handheld FastSCAN laser scanner (Polhemus, VT, USA). Digital fiducial points were recorded at five points on the individual’s head: the nasion, anterior of the left and right auditory canal, and three points on the forehead. Marker coils were placed at the same five positions to localize that person’s skull relative to the MEG sensors. The measurements of these marker coils were recorded both immediately prior and immediately after the experiment to correct for movement during the recording. All participants had less than 30mm of marker movement from the beginning of the experiment to the end of both tasks.

Before beginning the task inside the MEG, participants completed a training session on a desktop computer in order to familiarize themselves with the experimental tasks and the controls. Participants were allowed to complete the practice tasks as many times as needed in order to feel comfortable with the task. Once ready to begin the task, participants were asked to lay in a supine position with their head inside the MEG. It was stressed to participants, especially to children, that it is very important to keep their head and bodies as still as possible for the entirety of the experiment. After ending the experiment, participants were compensated monetarily or in class credit. Child participants were given a science-related toy or activity as reward for completing the experiment. The MEG measurements lasted roughly 45mins.

### MEG data acquisition and preprocessing

MEG data were collected in the Neuroscience of Language Lab in NYU New York using a whole-head 157 channel axial gradiometer system (Kanazawa Institute of Technology, Kanazawa, Japan). For the entirety of the experiment, participants lay in a dimly lit, magnetically shielded room. Vocal responses were captured with an MEG compatible microphone (Optimic 1160-FB).

MEG data were recorded at a sampling rate of 1000Hz (200Hz low-pass filter). The data was noise reduced by exploiting eight magnetometer reference channels located away from the participants’ heads via the Continuously Adjusted Least-Squares Method (CALM in the MEG Laboratory software (Yokogawa Electric Corporation and Eagle Technology Corporation, Tokyo, Japan)). The noise-reduced MEG recording, the digitized head-shape and the sensor locations were then imported into MNE-Python [42]. Our data analysis was time-locked to speech onset and then epoched 500ms backwards towards the picture onset, as we anticipated high variance in children’s response times and wanted to ensure that our epochs captured at least somewhat parallel processing stages across participants. While the two tasks were matched at articulation (that is, the same utterances were planned in both tasks), they were not matched in early parts of the trial (different pictures, different engagement of auditory processing), and thus speech onset was the natural point for time-locking our analysis.

Our artifact rejection routine consisted of (1) a low pass filter of 40 Hz, (2) independent component analysis to eliminate physiological artifacts that would contaminate our data of interest (i.e., blinks, heartbeats, and articulation artifacts), and (3) removing individual epochs that had amplitudes exceeding 2500 fT for any sensor at any time. One experimenter visualized each individual epoch and rejected any epochs that contained a sudden increase in the magnitude of the signal, as an increased number of motor artifacts in children meant that the automatic threshold was typically not sufficient to eliminate epochs containing extreme noise. This rejection routine resulted in the exclusion of 7.8% of trials (SD = 5.21) for the simple picture naming task, and 19.6% of trials (SD = 14.03) for the interactive naming task. Crucially, the combination of the epochs averaged backwards from the signal time-locked to speech onset and our strict artifact rejection allowed us to obtain spatiotemporal maps of the response-planning activity elicited by the picture, uncontaminated by motor or articulation artifacts.

Neuromagnetic data were coregistered with age-appropriate MRI templates [43, 44] by scaling the size of the average brain to fit the participant’s head-shape, aligning the fiducial points, and conducting final manual adjustments to minimize the difference between the head shape and the FreeSurfer average skull for adults and the NIHPD template for participants under 18 [43, 44]. Data were averaged for each task for each participant. The averages were low pass filtered at 40 Hz and high pass filtered at 1 Hz. The MEG facility on the New York University campus necessitated this strict high pass filter due to environmental nose. In both tasks, the period of 100 milliseconds before picture onset was used for baseline correction and the noise covariance matrix. Next, an ico-4 source space was created, consisting of 2562 potential electrical sources per hemisphere. At each source, activity was computed for the forward solution with the Boundary Element Model (BEM) method, which provides an estimate of each MEG sensor’s magnetic field in response to a current dipole at that source. The inverse solution was computed from the forward solution and the grand average activity across all trials, which determines the most likely distribution of neural activity. The resulting minimum norm estimates of neural activity [45] were transformed into normalized estimates of noise at each spatial location, resulting in statistical parametric maps (SPMs), which provide information about the statistical reliability of the estimated signal at each location in the map with millisecond accuracy. The SPMs were then converted to dynamic maps (dSPM). To quantify the spatial resolution of these maps, the pointspread function for different locations on the cortical surface was computed, which reflects the spatial blurring of the true activity patterns in the spatiotemporal maps, thus obtaining estimates of brain electrical activity with the best possible spatial and temporal accuracy [46]. The inverse solution was applied to each trial employing a fixed orientation of the dipole current that estimates the source normal to the cortical surface and retains dipole orientation.

### Data analysis

#### Behavioral data

*Reaction time*. Reaction time in the naming task was measured from the onset of the picture in simple picture naming and from the offset of the computer’s (i.e., “interlocutor’s”) speech in the interactive task. This way, the reaction times were expected to reflect the participants’ ability to begin planning their utterance already during the computer’s speech, as is natural in conversation, whereas in simple picture naming, no advance planning was possible. Despite the possibility of advance planning in the interactive task, participants were asked to not interrupt their interlocutor (the computer) and all trials in which they did were removed from all analyses. Trials with erroneous responses and those with response times above or below 2.5 standard deviations within each subjects’ data were also excluded from behavioral and MEG analyses. Log-transformed reaction time data were submitted to a linear mixed effects model (lmer) to assess significance of task and age differences.

*Accuracy*. Two research assistants individually listened to all responses. Trials with erroneous responses were not included in the analysis. Responses considered incorrect were trials in which the participant named the wrong object or color. Trials including lexical errors, such as saying “cup” instead of “glass” were not considered errors. Furthermore, perceptual errors such as naming the wrong color, “yellow” instead of “green”, were also excluded. We used generalized linear mixed effect model (glmer) for analyzing accuracy data.

For both reaction time and accuracy, Age Group (Adults vs. Teenagers vs. Children), Task (simple picture naming, interactive picture naming), Type (noun vs. phrase) and their interactions were treated as fixed factors in the models. The first level of each factor listed above was set as reference level. By-participant and by-item random intercepts, along with by-participant random slopes for the effects of Task and Type, were included in the models. We performed all the behavioral analyses in R (v4.0.2) [47]. The lme4 (v1.1.25) [48] and lmerTest (v3.1.3) [49] packages were used for linear-mixed effect modeling. For post-hoc comparison, emmeans (v1.5.2.1) [50] and phia (v0.2.1) [51] package was used for the analysis.

#### MEG data

For the statistical analysis of the MEG data, each age group (adults, teens, and children) was analyzed separately using eelbrain package (v0.35) [52]. Within each group, data for each time point were submitted to a full-hemisphere 2 x 2 spatiotemporal cluster permutation ANOVA with Task (Simple vs. Interactive picture naming) and Utterance Type (One-word vs. Two-word-phrase) as factors. Because our two tasks were cognitive at articulation and during articulation planning (since the same utterances were planned in both tasks), but differed substantially in the early parts of the trials (since the context manipulating took place during the first half of trials), our analysis focused on late stages of processing before articulation, starting at 500ms before speech onset and ended at speech onset (-500ms– 0ms). Following the analysis pipeline from previous MEG studies, datapoints were grouped into spatiotemporal clusters with cluster forming criteria of 25 spatial and 20 temporal points (= 20 ms) and alpha of .05 [53, 54]. The spatiotemporal clusters were entered into a permutation test of 10000 permutations of condition to yield corrected cluster p-values (alpha < 0.05) [55]. The left and right hemispheres of the brain were analyzed separately. Utterance type (One-word vs Two-word-phrase) had no significant effects within our analysis window and thus our results section focusses on the task effects only, the main topic of this report.

## Results

### Behavioral results

**Fig 2:**
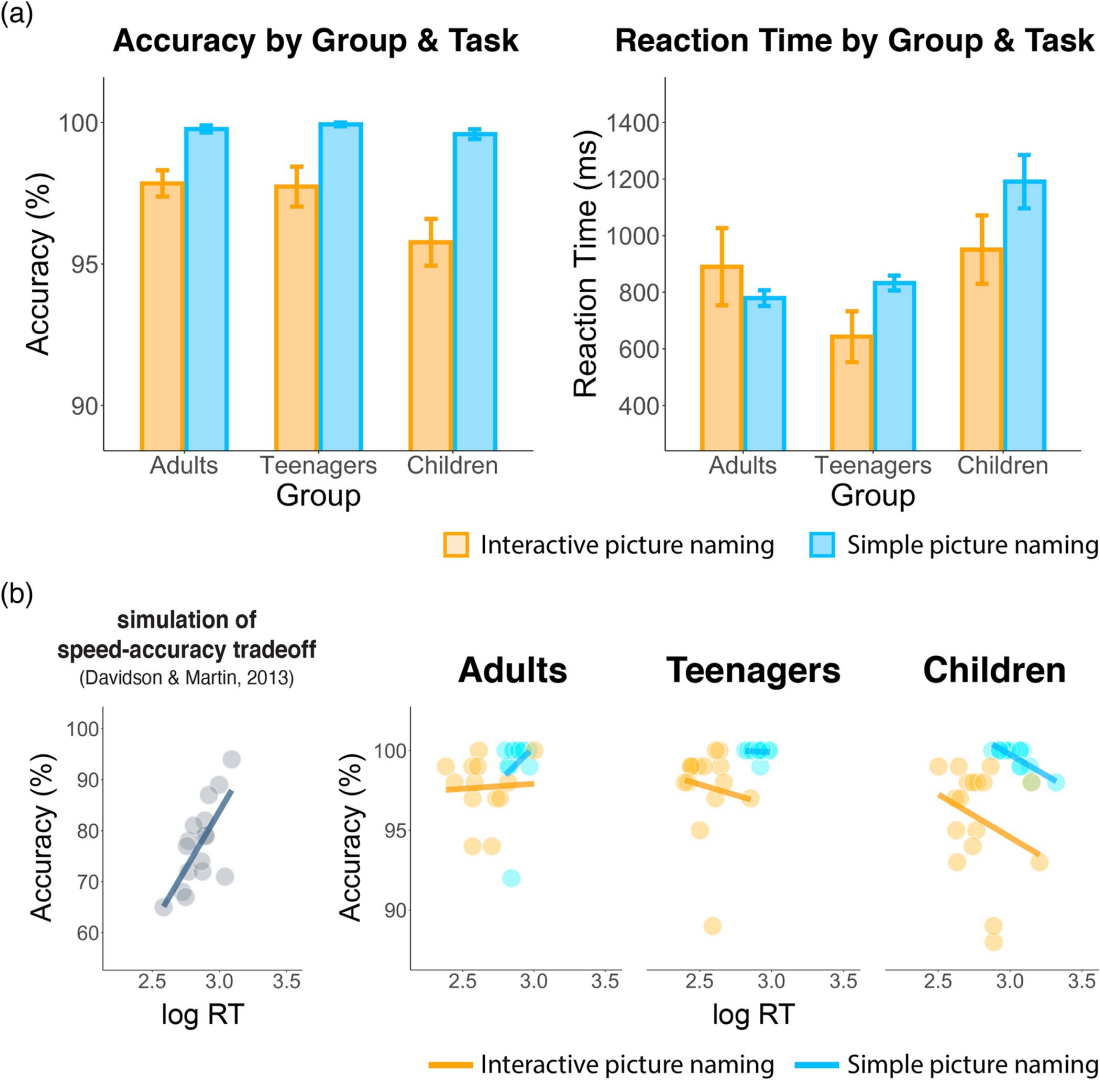
Behavioral results. (a) Accuracy and reaction time in both tasks for each group. Error bars represent the standard error of the mean (SEM). Overall, participants exhibited faster responses in the interactive picture naming task compared to the simple picture naming task. Children had slower responses compared to adults, whereas there was no difference in response time between teenagers and adults. Accuracy was overall high, showing no main effects or interactions. (b) Scatter plots of speed-accuracy tradeoff from the simulation in Davidson & Martin [56] and from the behavioral performance in each group. No evidence of speed-accuracy tradeoffs was observed. S1 File contains tables for the full behavioral results.

Behavioral data from four adults and one child were missing due to a technical problem during the experiment. The remaining behavioral data from 45 participants (13 adults, 15 teenagers, and 17 children) were used for the following analyses. Fig 2 depicts the means and standard errors of our reaction time and accuracy data. Reaction time in the naming task was measured from the onset of the picture in simple picture naming and from the offset of the computer’s (i.e., “interlocutor’s”) speech in the interactive task.

#### Reaction time

Participants’ data were analyzed using the lmer model: *log*_*10*_*(Reaction time) ~ Age Group × Task × Type + (1 + Task + Type | Participant) + (1 | Item)*. To examine the significance of main effects and interaction, likelihood-ratio tests were used by comparing a full model with a reduced model. Overall, participants exhibited faster responses in the interactive picture naming task compared to the simple picture naming task [*β*_interactive_ = -0.28, *SE* = 0.04, χ^2^(1) = 68.65, *p* < .001] as well as faster responses for nouns than phrases [*β*_phrase_ = 0.05, *SE* = 0.02, χ^2^(1) = 12.04, *p* < .001]. Additionally, there was a significant main effect of Age Group [χ^2^(2) = 26.27, *p* < .001]. Further pairwise comparison showed that children had slower responses compared to adults [*β* = 0.14, *SE* = 0.03, *z* = 3.93, *p* < .001], whereas there was no difference in response time between teenagers and adults [*β* = 0.02, *SE* = 0.03, *z* = 0.77, *p* = .719]. Overall, the variance in reaction time was larger in the interactive than in the simple picture naming task, most likely due to the higher complexity of the interactive task.

A significant three-way interaction of Age group, Task, and Type was observed in reaction time [χ^2^(11) = 143.43, *p* < .001]. Further analyses of the interaction revealed that, in the simple naming task, participants from all age groups displayed faster responses to nouns than phrases [*ps* = 1]. In the interactive picture naming task, adults exhibited a similar pattern of faster responses to nouns than phrases, but teenagers and children did not, resulting in a significant difference between both the adult and the teenager pattern [χ^2^(1) = 16.44, *p* < .001] and the adult and the child pattern [χ^2^(1) = 10.63, *p* = .006]. That is, both teenagers and children showed similar reaction times for nouns and phrases.

#### Accuracy

Participants’ data were analyzed using the glmer model: *Accuracy ~ Age Group* × *Task* × *Type + (1 + Task + Type | Participant) + (1 | Item)*. As shown in Fig 2A, all age groups performed with high accuracy in both naming tasks. No main effects or interactions were observed in the model. Furthermore, we examined if our results contained speed-accuracy tradeoffs (Fig 2B) using the approach by Davidson & Martin [56]. The glmer model for speed-accuracy tradeoff was constructed as follows: *Accuracy ~ log*_*10*_*(Reaction Time)* × *Age Group* × *Task* × *Type + (1 + Task + Type + log10(Reaction Time)) | Participant) + (1 | Item)*. No main effects or interactions were found, indicating that none of the age groups demonstrated a speed-accuracy tradeoff in either of the naming tasks.

### MEG results

**Fig 3:**
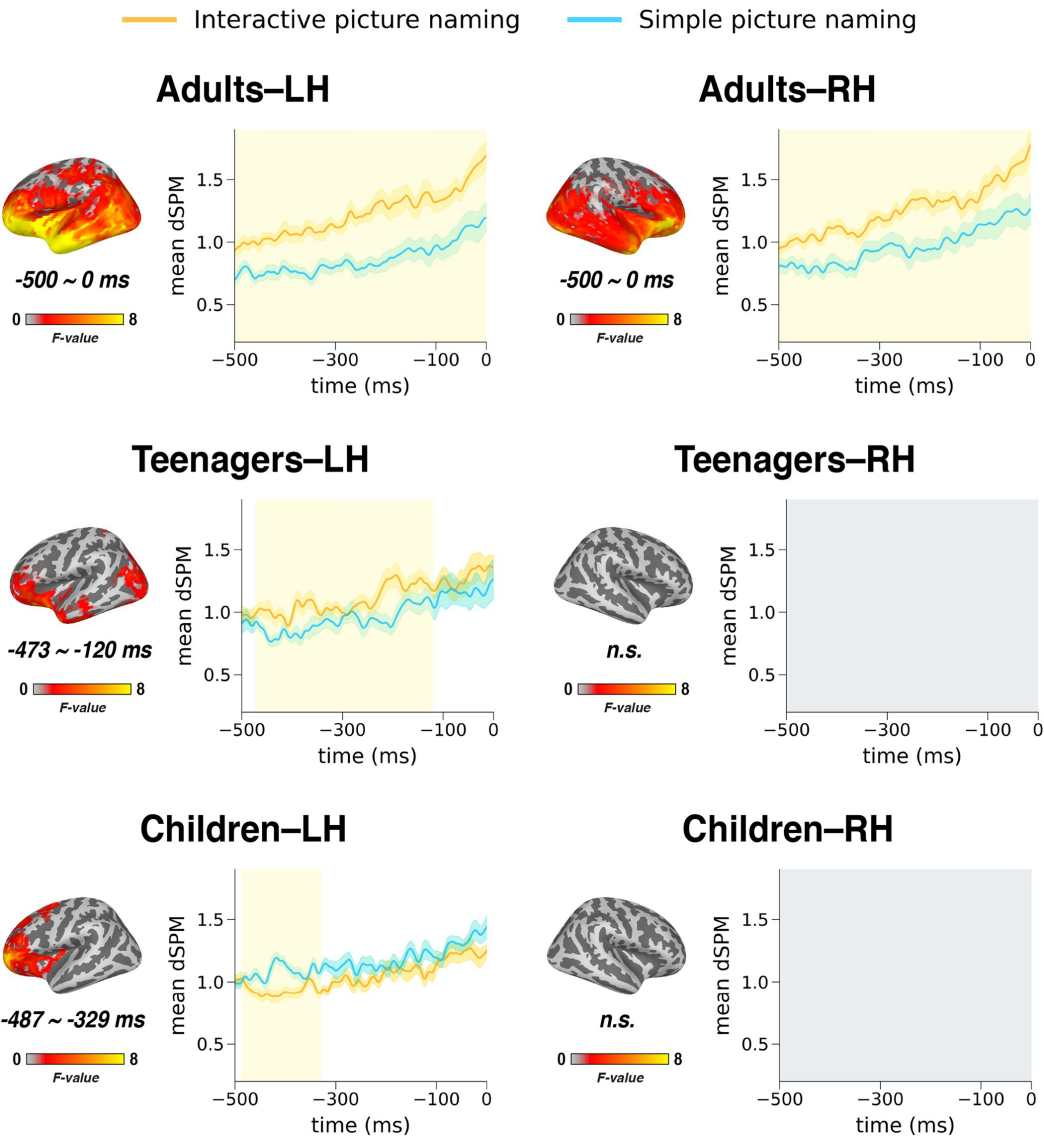
MEG task effects. Main effect of task from the spatiotemporal cluster-based permutation tests for the left and right hemispheres in adults, teenagers and children (yellow shading indicates p < .05 corr.). In the left hemisphere, adults (N = 17, *M*_*age*_ = 35) showed a robust bilateral activity increase for the interactive context over the non-interactive context in the entire temporal lobe and extending into inferior frontal and parietal cortex as well as the occipital lobe. In teenagers (N = 15, *M*_*age*_ = 18), the left anterior temporal and left inferior frontal and occipital cortices also showed an increase for the interactive task, but the right hemisphere showed no task effect. In children (N = 18, *M*_*age*_ = 9), we observed the reverse pattern: an increase for simple picture naming over interactive picture naming left laterally in frontal cortex. LH: left hemisphere. RH: right hemisphere.

**Fig 4:**
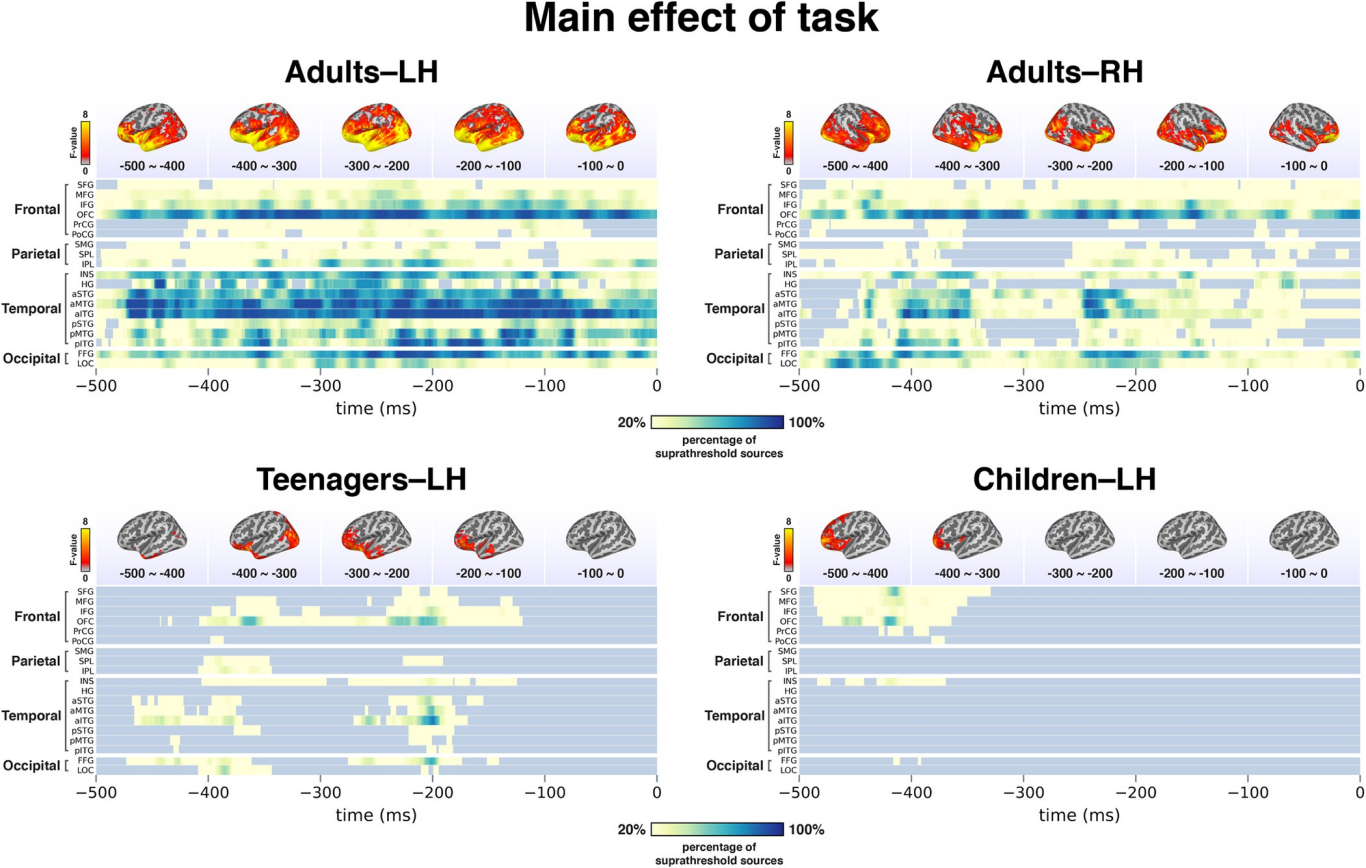
Decomposition of spatiotemporal clustering results. The large spatiotemporal clusters shown in Fig 3 are decomposed into their subparts here, with the top panels showing the mean F-values of the task effects averaged across 100 ms intervals and the bottom panels plotting heat maps of the spatiotemporal profiles of the clusters, indicating the percentage of suprathreshold sources (vertices with F-values higher than the statistical threshold) in brain regions from -500 ms to 0 ms before speech onset. The adult pattern is a strong engagement of orbitofrontal (OFC) and temporal cortices in particular. The patterns is weakly seen in teenagers. Children also show OFC involvement, but as depicted in Fig 3, the effect directionality is reverse for children as compared to adults and teenagers, that is, higher activity for simple picture naming.

Our bilateral full-hemisphere analysis provided a comprehensive spatiotemporal characterization of the effect of our context manipulation, as shown in Figs 3 and 4. Fig 3 plots the significant spatiotemporal clusters and their timecourses. The timecourse waveforms plot the dynamics of the entire spatial cluster, with all sources within the cluster averaged together. In Fig 4, we show a decomposition of the rather large clusters identified by the spatiotemporal clustering analysis, plotting the relative contributions of different regions at different times.

In adults, the interactive context elicited a robust signal increase in the left hemisphere throughout the analysis window, starting at 500ms before speech onset until speech onset (*p* < .001, *t*_*mi*n_ = -500ms, *t*_*max*_ = 0ms, cluster size: 2363 vertices). The increase covered the entire left perisylvian language network, especially for the orbitofrontal cortex (OFC) and the anterior portion of temporal lobe. A similar pattern was observed in the right hemisphere (*p* < .001, *t*_*min*_ = -500ms, *t*_*max*_ = 0ms, cluster size: 2047 vertices), though it was somewhat less pronounced in the temporal cortex. Thus, in adults the interactive context engaged most areas of the brain more than non-interactive naming, with only motor cortex and its adjacent regions not being impacted. Critically, motor planning was controlled between the two tasks, that is, both tasks elicited the same utterances. The sustained duration of the effect suggests an “interactive mode” rather than more specific transient computations.

In teenagers, a similar pattern to the adults was observed in the left hemisphere, though it was spatially less expansive: left anterior temporal and left prefrontal cortices showed an increase for the interactive task, as did areas of occipital cortex (*p* = .002, *t*_*min*_ = -473ms, *t*_*max*_ = -120ms, cluster size = 1168 vertices). Like in adults, the effect was sustained, though it did start shortly after the beginning of the analysis window. However, unlike adults, teenagers showed no task effect in the right hemisphere and thus, only their left hemisphere was adult-like.

Finally, the children’s profile was very different: instead of activity increases for the interactive context, we found significant effects only in the other direction, that is, increases for simple naming. The distribution of the effect was purely frontal, not temporal, only in the left hemisphere (*p* = .032, *t*_*min*_ = -487ms, *t*_*max*_ = -330ms, cluster size = 803 vertices).

Although the children’s reaction times were rather uniformly longer than our 500ms analysis epoch, this was not the case for 3 adults and 7 teens, whose mean reaction times in the interactive task were shorter than 500ms. Since the adults’ and teens’ results were sustained and lasted up until or close to speech onset, their results are unlikely to be driven by the auditory signal, which would have only been audible for ~100-200ms of the start of the trial. Also, that since simultaneous listening and the speech planning are very much essence of interaction, in a sense the auditory “confound” is a core part of our intended manipulation. Nevertheless, reran all the analyses without the subjects with fast reaction times (M < 500ms), and replicated all results: for adults (N = 14), the sustained, broadly distributed bilateral fronto-temporal clusters were again observed for the whole analysis window (LH: *p* < .001, *t*_*min*_ = -500ms, *t*_*max*_ = -0ms, cluster size = 2358 vertices; RH: *p* < .001, *t*_*min*_ = -500ms, *t*_*max*_ = -0ms, cluster size = 1990 vertices) and for teens (N = 8), we observed a less spatially distributed frontotemporal cluster similar to the main analysis in the left hemisphere only (LH: *p* < .001, *t*_*min*_ = -496ms, *t*_*max*_ = -0ms, cluster size = 1292 vertices). One child participant also had a faster than 500ms utterance time mean for the interactive task. Removing this participant replicated the LH activity increase for simple picture naming in two separate clusters, both with a frontal distribution like in the main analysis (LH cluster 1: *p* < .001, *t*_*min*_ = -497ms, *t*_*max*_ = -146ms, cluster size = 1557 vertices; LH cluster 2: *p* < .001, *t*_*min*_ = -193ms, *t*_*max*_ = -0ms, cluster size = 534 vertices), which is statistically a stronger result than the original analysis. Thus removing the quickly responding participants either yielded almost identical results to the main analysis (adults and teens) or a stronger version of the pattern seen in the main analysis.

## Discussion

In this work we sought to characterize differences in neural activity elicited by simple picture naming versus our novel interactive picture naming task. We assessed participants of three different age groups: children, teenagers, and adults, to observe developmental changes as related to language production in these two distinct language production situations.

Our MEG data revealed a relatively straightforward developmental trajectory from childhood, to adolescence, into adulthood. Adults showed a robust, sustained, bilateral activity increase for the interactive task as compared to simple picture naming. In teenagers, this pattern was present in the left hemisphere, but not in the right. In children, neither hemisphere was adult like. Instead, children showed an activity increase for the simple picture naming task in left lateral frontal cortex. The effect was only observed in the first half of the analysis window, that is, it did not persist up until articulation like the activity increases in teens and adults. In sum, the results suggest a faster maturation of the left than the right hemisphere, since the LH of teens was adult-like but not the RH, and a generally slow emergence of interaction-related activity increases. The children’s activity increase in left inferior frontal cortex for simple picture naming conforms to prior picture naming results in healthy children, implicating the left IFG [26, 29]. Also, although the activity increase elicited by interaction in adults was very broadly distributed, we see a particularly clear and sustained activation of the OFC, including in the teens (Fig 4). This is consistent with prior work implicating the medial prefrontal cortex for social cooperation in conversational contexts [31–33]. This suggests that our highly controlled interaction with the computer may, in fact, have tapped neural processes that operate in interactions with humans as well. Thus, our findings can inform future research aimed at understanding the connection between human-to-human and human-to-machine interactions, particularly as the latter continues to gain prominence in the AI era. Our decision to opt for computer interaction was, however, driven solely by implementational simplicity: we wanted to offer a protocol that would be maximally easy to use in a variety of settings, including clinical ones.

We expected the contextual support afforded by the interactive task to manifest as faster reaction times than simple picture naming times, as measured from the computer’s speech offset in the interactive task and from picture onset in the simple naming task. This main effect was observed, though interestingly, it was only apparent in the children’s and teenagers’ RT means, and not in the adults’ (Fig 2, though note that the interaction between task and age group was not significant). Thus it appeared that the children and teenagers benefited more than the adults from the possibility to proactively plan their utterances in the interactive task.

In light of our hypothesized context-driven activity reductions and interaction-driven activity increases, the following overall picture is suggested by the combination of our behavioral and neural data: the children’s data uniformly conform to contextual facilitation in the interactive task, while the adults’ data exhibit robust activity increases potentially as a function of the additional task components of interaction. Since adults’ behavioral data show no clear speed-up for the interactive task, the faciliatory effect of context may have been overridden by these added task components. According to our findings, an adult-like interactive brain matures very slowly, since although our teens’ left hemispheres were adult-like, their right hemispheres were not.

While we find this the most parsimonious account of the relationship between the neural and behavioral data, it is by no means the only possibility. For example, the children’s signal increases for simple picture naming could reflect higher competition among possible utterances, since no context is presented in this task to narrow down the search space. The plausibility of such an account is enhanced by the frontal localization of the effect, with peak activity in left inferior frontal cortex, implicated for selection demands among alternatives [57]. Thus future work should directly pit contextual facilitation and lexical competition against each other as hypotheses.

Overall, we suggest that the sustained frontal-temporal activity of the interactive task that begins to emerge in adolescence may reflect a rather global “interactive mode” brain state, indexing many processes and representations of conversational alignment and turn-taking [2, 58]. While the simple set-up of this study does not yet allow for more detailed functional interpretations, our robust task effects offer an opportunity for future research to unpack them more mechanistically. This should include assessing whether interaction with a computer, as implemented here for maximal simplicity, is representative of interaction with a human.

In sum, by comparing classic picture naming with a more interactive version of the task, we identified a broad sustained activation pattern that emerged in the left hemisphere in teenagers and became strongly bilateral by adulthood. Although arising from a highly controlled laboratory task, these findings suggest that an adult-like interactive brain is slow to develop.

## Supporting information

S1 File Summaries of the linear mixed effect model for reaction time (RT) and the generalized linear mixed effect models for accuracy (ACC) and for speed-accuracy tradeoffs. (DOCX)
