# Supplementary material for: MEG correlates of speech planning in simple vs. interactive picture naming in children and adults
Source: PLoS One. 2023 Oct 17;18(10):e0292316. doi: 10.1371/journal.pone.0292316 (PMC10581494; doi:10.1371/journal.pone.0292316)
Supplement: S1 File — (DOCX) [file pone.0292316.s001.docx]

**Supplementary tables**

**Table S1.** Summary of linear mixed effect model for reaction time (RT)

| Formula: log_10_(RT) ~ Group × Task × Type + (1 + Task + Type \| Participant) + (1 \| Item) | | | | |
| --- | --- | --- | --- | --- |
| Fixed effects: | *β* | SE | *t* value | *p* |
| (intercept) | 2.91 | 0.01 | 223.86 | .000 |
| Group [Teenagers] | -0.03 | 0.02 | -1.64 | .108 |
| Group [Children] | 0.09 | 0.02 | 4.78 | .000 |
| Task | -0.27 | 0.02 | -12.52 | .000 |
| Type | 0.06 | 0.01 | 4.30 | .000 |
| Group [Teenagers] × Task | -0.05 | 0.03 | -1.47 | .147 |
| Group [Children] × Task | 0.05 | 0.03 | 1.63 | .109 |
| Group [Teenagers] × Type | 0.00 | 0.02 | -0.13 | .900 |
| Group [Children] × Type | 0.02 | 0.02 | 0.80 | .426 |
| Task × Type | -0.02 | 0.01 | -1.48 | .140 |
| Group [Teenagers] × Task × Type | -0.06 | 0.02 | -3.25 | .001 |
| Group [Children] × Task × Type | -0.04 | 0.02 | -2.46 | .014 |
| Random effects: | Variance |  |  |  |
| Participant | 0.00 |  |  |  |
| Item | 0.01 |  |  |  |

**Table S2.** Summary of generalized linear mixed effect model for accuracy (ACC)

| Formula: ACC ~ Group × Task × Type + (1 + Task + Type \| Participant) + (1 \| Item) | | | | |
| --- | --- | --- | --- | --- |
| Fixed effects: | *β* | SE | *z* value | *p* |
| (intercept) | 17.70 | 14.77 | 1.20 | .231 |
| Group [Teenagers] | 5.31 | 15.23 | 0.35 | .727 |
| Group [Children] | 5.17 | 15.99 | 0.32 | .746 |
| Task | -13.53 | 14.77 | -0.92 | .360 |
| Type | -11.15 | 14.77 | -0.76 | .450 |
| Group [Teenagers] × Task | -5.13 | 15.23 | -0.34 | .736 |
| Group [Children] × Task | -6.23 | 15.99 | -0.39 | .697 |
| Group [Teenagers] × Type | -4.15 | 15.23 | -0.27 | .785 |
| Group [Children] × Type | -5.40 | 15.99 | -0.34 | .736 |
| Task × Type | 11.63 | 14.77 | 0.79 | .431 |
| Group [Teenagers] × Task × Type | 4.72 | 15.23 | 0.31 | .757 |
| Group [Children] × Task × Type | 5.70 | 15.99 | 0.36 | .722 |
| Random effects: | Variance |  |  |  |
| Participant | 0.05 |  |  |  |
| Item | 1.44 |  |  |  |

**Table S3.** Summary of generalized linear mixed effect model for speed-accuracy tradeoffs

| Formula: ACC ~ log_10_(RT) × Group × Task × Type + (1 + Task + Type + log_10_(RT) \| Participant) + (1 \| Item) | | | | |
| --- | --- | --- | --- | --- |
| Fixed effects: | *β* | SE | *z* value | *p* |
| (intercept) | -1.16 | 26.84 | -0.04 | .966 |
| log_10_(RT) | 6.90 | 26.74 | 0.26 | .796 |
| Group [Teenagers] | 20.82 | 28.09 | 0.74 | .459 |
| Group [Children] | 21.78 | 32.60 | 0.67 | .504 |
| Task | 8.57 | 26.86 | 0.32 | .750 |
| Type | 12.01 | 27.06 | 0.44 | .657 |
| log_10_(RT) × Group [Teenagers] | -5.49 | 26.85 | -0.20 | .838 |
| log_10_(RT) × Group [Children] | -5.79 | 27.51 | -0.21 | .833 |
| log_10_(RT) × Task | -8.07 | 26.74 | -0.30 | .763 |
| Group [Teenagers] × Task | -19.48 | 28.10 | -0.69 | .488 |
| Group [Children] × Task | -21.15 | 32.62 | -0.65 | .517 |
| log_10_(RT) × Type | -8.25 | 26.76 | -0.31 | .758 |
| Group [Teenagers] × Type | 6.48 | 28.55 | 0.23 | .821 |
| Group [Children] × Type | -24.48 | 32.87 | -0.75 | .456 |
| Task × Type | -11.05 | 27.09 | -0.41 | .683 |
| log_10_(RT) × Group [Teenagers] × Task | 5.02 | 26.84 | 0.19 | .852 |
| log_10_(RT) × Group [Children] × Task | 5.26 | 27.51 | 0.19 | .848 |
| log_10_(RT) × Group [Teenagers] × Type | -3.29 | 26.91 | -0.12 | .903 |
| log_10_(RT) × Group [Children] × Type | 6.54 | 27.63 | 0.24 | .813 |
| log_10_(RT) × Task × Type | 8.10 | 26.77 | 0.30 | .762 |
| Group [Teenagers] × Task × Type | -4.29 | 28.58 | -0.15 | .881 |
| Group [Children] × Task × Type | 22.62 | 32.91 | 0.69 | .492 |
| log_10_(RT) × Group [Teenagers] × Task × Type | 2.72 | 26.92 | 0.10 | .920 |
| log_10_(RT) × Group [Children] × Task × Type | -5.80 | 27.64 | -0.21 | .834 |
| Random effects: | Variance |  |  |  |
| Participant | 0.04 |  |  |  |
| Item | 0.50 |  |  |  |
